# Epigenetic regulation of gene expression in rare inherited retinal disorders

**DOI:** 10.3389/fgene.2026.1806258

**Published:** 2026-03-02

**Authors:** Feliciana Menna, Laura De Luca, Alessandro Meduri, Antonio Baldascino, Stefano Lupo, Enzo Maria Vingolo

**Affiliations:** 1 Department of Medical-Surgical Sciences and Biotechnologies, U.O.C. Ophthalmology, Sapienza University of Rome, Rome, Italy; 2 Department of Biomedical and Dental Science and of Morphological and Functional Images, University of Messina, Messina, Italy; 3 Ophthalmology Unit, Fondazione Policlinico Universitario A. Gemelli IRCCS, Rome, Italy

**Keywords:** chromatin remodeling, DNA methylation, epigenetics, inherited retinal diseases, non-coding RNA, photoreceptors, retina

## Abstract

The retina is a highly specialized neural tissue characterized by extreme cellular differentiation, high metabolic demand, and lifelong exposure to environmental stressors. These features render retinal cell identity exquisitely dependent on epigenetic regulation of gene expression. Rare hereditary retinal disorders offer a unique framework for understanding how epigenetic mechanisms modulate genotype–phenotype relationships in the human eye. This Mini Review provides an integrated overview of DNA methylation, histone modifications, chromatin remodeling, and non-coding RNA-mediated regulation in retinal development, homeostasis, and degeneration. We discuss how epigenetic dysregulation contributes to photoreceptor loss, phenotypic variability, and disease progression in inherited retinal dystrophies and syndromic disorders affecting the retina, and we highlight emerging translational opportunities and current limitations of epigenetic-based therapeutic strategies for rare retinal disease.

## 1 Introduction

The retina is a laminated extension of the central nervous system that converts light into neural signals through highly specialized photoreceptors and interneurons. Establishing and maintaining this architecture requires precise, cell-type–specific control of gene expression from embryogenesis through adult life. Epigenetic mechanisms—including DNA methylation, histone post-translational modifications, ATP-dependent chromatin remodeling, and non-coding RNAs—provide this control by shaping chromatin accessibility and transcriptional competence without changing DNA sequence (Raeisossadati et al., 2021; Aldiri and Vetter, 2012; Iwagawa and Watanabe, 2019).

Inherited retinal diseases (IRDs) such as retinitis pigmentosa (RP), cone–rod dystrophy (CRD), and Leber congenital amaurosis (LCA) display striking clinical heterogeneity, even among individuals carrying the same pathogenic variant. Differences in age of onset, progression rate, and regional vulnerability suggest that epigenetic context can modulate genotype–phenotype relationships. Here, we summarize major epigenetic mechanisms relevant to retinal development and degeneration, provide disease-oriented examples from IRDs and syndromic disorders, and critically discuss translational opportunities and limitations for epigenetic-based interventions.

## 2 Epigenetic mechanisms shaping retinal gene expression

Four interrelated layers of regulation dominate current IRD-focused epigenetic literature: DNA methylation, histone modifications, chromatin remodeling, and non-coding RNAs. Although they are often discussed separately, they function as an integrated system coordinating developmental programs, long-term neuronal identity, and stress responses.

### 2.1 DNA methylation in retinal development and inherited degeneration

During retinal neurogenesis, progressive demethylation at photoreceptor-specific promoters and enhancers enables activation of lineage-defining transcription factors such as OTX2, CRX, and NRL (Dvoriantchikova et al., 2019; Mo et al., 2016). TET-dependent active demethylation contributes to opening cis-regulatory elements that control photoreceptor identity (Dvoriantchikova et al., 2019). In experimental systems, disrupting these methylation dynamics impairs activation of downstream phototransduction genes, supporting a developmental contribution to early-onset dystrophies.

Maintenance methylation is also essential for retinal homeostasis. Conditional loss of DNMT1 compromises survival of post-mitotic retinal neurons and leads to laminar disruption and photoreceptor degeneration (Rhee et al., 2012). In several preclinical models of retinal degeneration, altered DNA methylation signatures emerge early—sometimes preceding overt photoreceptor death—suggesting that methylation changes can act as upstream amplifiers of cellular stress (Dvoriantchikova et al., 2019; Wahlin et al., 2017). Disease-relevant examples include methylation-associated repression of metabolic and antioxidant programs that may exacerbate degeneration triggered by pathogenic variants in photoreceptor genes (e.g., RHO- or RPGR-associated RP) by reducing energetic reserve and stress-buffering capacity (Wahlin et al., 2017; Sancho-Pelluz et al., 2010; Punzo et al., 2009; Dyer and Cepko, 2001).

Human retinal epigenomic data remain limited because of restricted tissue availability. Accordingly, much of the mechanistic evidence in IRDs comes from animal models, retinal explants, and patient-derived organoids, which may not fully recapitulate adult human retinal architecture and microenvironment (Wang et al., 2022; Liang et al., 2023; Finkb et al., 2022).

### 2.2 Histone modifications and chromatin remodeling

Histone acetylation and methylation dynamically tune chromatin accessibility at promoters and enhancers of retinal genes, thereby modulating transcriptional output. During photoreceptor differentiation, permissive acetylation states support activation of phototransduction, synaptic, and retinoid-cycle gene networks downstream of core transcription factors. HDAC1 contributes to establishing appropriate acetylation patterns during rod maturation (Chen and Cepko, 2007; Ferreira et al., 2017).

In degenerating retinas, however, increased or dysregulated HDAC activity has repeatedly been linked to photoreceptor death pathways. In RP models, HDAC activation is detected early—before caspase-dependent apoptosis—consistent with chromatin dysregulation as a driver rather than a bystander event (Sancho-Pelluz et al., 2010). Pharmacologic HDAC inhibition (e.g., trichostatin A) has been reported to preserve cones in mouse models of RP, highlighting a mutation-independent, pathway-convergent therapeutic rationale (Trifunović et al., 2016).

ATP-dependent chromatin remodelers and histone-modifying enzymes also contribute to retinal vulnerability through higher-order genome organization and enhancer–promoter communication. Syndromic disorders caused by pathogenic variants in epigenetic regulators—such as Rubinstein–Taybi syndrome (CREBBP/EP300) and Kabuki syndrome (KMT2D/KDM6A)—illustrate how global chromatin imbalance can produce variable retinal phenotypes (Bjornsson, 2015; Lacombe et al., 2024; Aref-Eshghi et al., 2017). These ‘natural experiments’ strengthen the concept that retina, and especially photoreceptors, are unusually sensitive to perturbations in chromatin accessibility and enhancer function.

### 2.3 Non-coding RNAs as post-transcriptional and epigenetic regulators

Non-coding RNAs provide a critical post-transcriptional layer that intersects with chromatin regulation by shaping the abundance of transcription factors and chromatin modifiers. MicroRNAs (miRNAs) are broadly required for retinal development and neuronal survival; loss of Dicer, a key enzyme for miRNA biogenesis, leads to widespread retinal degeneration (Reh and Hindges, 2018; Georgi and Reh, 2010). A well-studied disease-relevant example is the retina-enriched miR-183/96/182 cluster, which regulates photoreceptor differentiation and survival and modulates stress-response pathways (Taylor et al., 2019).

In inherited degeneration, dysregulated miRNA networks can exacerbate oxidative stress, inflammation, and apoptosis, thereby modifying severity across IRDs (Taylor et al., 2019; Karali et al., 2010; Pawlick et al., 2021). For instance, in ABCA4-associated disease, impaired retinoid handling increases toxic by-product accumulation; altered post-transcriptional control of oxidative-stress and inflammatory pathways may further accelerate cone loss (Taylor et al., 2019; Karali et al., 2010; Pawlick et al., 2021). Long non-coding RNAs are increasingly implicated in retinal development and disease, but mechanistic data in IRDs remain comparatively sparse, underscoring a need for functional validation in human-based systems.

## 3 Epigenetic modifiers of phenotypic variability in inherited retinal disease

A hallmark of IRDs is variable penetrance and expressivity among individuals sharing the same causal variant. Epigenetic context can shape transcriptional competence, metabolic adaptability, and stress-response capacity, thereby amplifying or attenuating disease phenotypes (Raeisossadati et al., 2021; Aldiri and Vetter, 2012; Iwagawa and Watanabe, 2019; Dvoriantchikova et al., 2019; Mo et al., 2016; Bjornsson et al., 2014). Below we highlight representative disease groups.

### 3.1 Retinitis pigmentosa: convergent epigenetic stress programs

RP is genetically heterogeneous, involving genes affecting phototransduction (e.g., RHO), ciliary trafficking (RPGR/RP2), extracellular structure (USH2A/EYS), and splicing factors (PRPF31/PRPF8) (Raeisossadati et al., 2021; Aldiri and Vetter, 2012). Despite distinct primary insults, many RP models converge on shared stress programs that include early chromatin remodeling and repression of metabolic homeostasis genes (Sancho-Pelluz et al., 2010; Punzo et al., 2009). This convergence supports a framework in which epigenetic alterations (HDAC activation, stress-associated methylation changes, and enhancer remodeling) operate downstream of diverse mutations and can influence both rod death and secondary cone degeneration.

Therapeutically, this suggests that epigenetic modulation could complement gene-specific approaches by stabilizing cellular stress responses and preserving transcriptional networks needed for survival. However, because these regulators act genome-wide, the balance between neuroprotection and off-target transcriptional disruption is a central translational concern (see Translational perspectives).

### 3.2 Cone–rod dystrophy: enhancer dependence of cone identity programs

CRDs (e.g., ABCA4-, CRX-, GUCY2D-, PROM1-, and RPGRIP1-associated disease) are characterized by primary cone dysfunction followed by secondary rod loss (Aldiri and Vetter, 2012; Swaroop et al., 2010). Cones rely on extensive enhancer and super-enhancer architectures to sustain high transcriptional output for photopic vision, synaptic signaling, and rapid metabolic turnover. This configuration may render cones particularly sensitive to disturbances in chromatin accessibility and enhancer–promoter communication.

Disease-relevant examples include perturbation of cone-enriched miRNA programs and stress-induced chromatin changes that reduce transcriptional robustness (Taylor et al., 2019; Swaroop et al., 2010; Busskamp et al., 2014). These mechanisms may partially explain interindividual variability in progression and differential responsiveness to gene-based interventions, even within the same genetic subgroup.

### 3.3 Leber congenital amaurosis: developmental programming and therapeutic timing

LCA presents in infancy and frequently involves genes expressed during retinal development (e.g., RPE65, CRB1, RDH12, CEP290, CRX). During neurogenesis, coordinated DNA demethylation, histone acetylation, and chromatin remodeling establish photoreceptor competence and synaptic maturation (Dvoriantchikova et al., 2019; Mo et al., 2016; Rhee et al., 2012). If early chromatin programs are disrupted, aberrant states may become relatively stable, limiting later transcriptional plasticity.

The clinical success of gene augmentation for RPE65-mediated LCA demonstrates that photoreceptors can retain functional capacity, yet outcomes vary substantially between patients (Russell et al., 2017). Epigenetic constraints—together with disease stage and residual cell number—may contribute to this variability and reinforce the importance of early intervention in developmental IRDs.

### 3.4 Syndromic retinal disorders: global chromatin imbalance with tissue-selective vulnerability

Syndromes caused by variants in the epigenetic machinery (e.g., CREBBP/EP300 in Rubinstein–Taybi; KMT2D/KDM6A in Kabuki) provide strong human-genetic support for the role of chromatin regulation in ocular development and maintenance (Bjornsson, 2015; Lacombe et al., 2024; Aref-Eshghi et al., 2017). Retinal involvement is variable, suggesting modulation by residual enzyme activity, compensatory pathways, and environmental exposures. These disorders reinforce that photoreceptors’ high transcriptional demand and post-mitotic status make them particularly vulnerable to chromatin dysregulation.

#### 3.4.1 Rubinstein–Taybi syndrome

Rubinstein–Taybi syndrome (RTS) is caused by heterozygous pathogenic variants in *CREBBP* or *EP300*, which encode histone acetyltransferases (HATs) responsible for depositing acetyl marks on histones H3 and H4 at active promoters and enhancers. These enzymes act as transcriptional co-activators for a broad range of developmental transcription factors and are essential for maintaining open chromatin states and transcriptional competence.

In the retina, impaired HAT activity leads to reduced histone acetylation at regulatory regions controlling genes involved in photoreceptor differentiation, synaptic transmission, and metabolic homeostasis. Given the high transcriptional demand of photoreceptors, even partial reductions in chromatin acetylation can compromise long-term cellular stability. Clinically, RTS patients may exhibit retinal dystrophy, coloboma, or progressive visual impairment, although penetrance and severity vary widely, suggesting modulation by additional epigenetic or environmental factors (Lacombe et al., 2024).

#### 3.4.2 Kabuki syndrome

Kabuki syndrome is most commonly caused by mutations in *KMT2D*, a histone methyltransferase responsible for H3K4 mono- and trimethylation, and less frequently in *KDM6A*, a histone demethylase that removes repressive H3K27 methylation marks. Together, these enzymes regulate the balance between transcriptionally active and repressed chromatin states at developmental gene loci.

Disruption of H3K4 methylation or aberrant persistence of repressive histone marks alters enhancer activation and compromises long-range chromatin interactions. In the retina, such defects can impair transcriptional programs required for retinal development, synaptic connectivity, and photoreceptor maintenance. Ocular manifestations in Kabuki syndrome include retinal dystrophy, optic nerve anomalies, and colobomatous defects, with marked interindividual variability reflecting differential epigenetic compensation and tissue resilience (Aref-Eshghi et al., 2017).

#### 3.4.3 Implications for genotype–phenotype relationships

The study of syndromic retinal disorders underscores the importance of epigenetic context in shaping genotype–phenotype relationships. In these conditions, retinal degeneration does not arise from disruption of a single retina-specific pathway, but from cumulative transcriptional instability affecting multiple cellular processes. This perspective reinforces the concept that epigenetic state acts as a critical modifier of disease severity and progression, even in disorders with a clear monogenic origin (Bjornsson, 2015; Lacombe et al., 2024; Aref-Eshghi et al., 2017).

## 4 Discussion - epigenetic heterogeneity and disease course in IRDs

Across IRDs, intercellular and interindividual differences in chromatin state—including promoter/enhancer accessibility, three-dimensional genome organization, and stress-response gene control—can modulate transcriptional output downstream of identical pathogenic variants (Sancho-Pelluz et al., 2010; Punzo et al., 2009). Recent single-cell multi-omic analyses of human retina and retinal organoids reveal marked cell-type–specific epigenomic architectures, with distinct enhancer repertoires in rods, cones, RPE, and Müller glia (Cowan et al., 2020; Wang et al., 2022; Liang et al., 2023; Finkb et al., 2022).

This heterogeneity is dynamic and influenced by environmental modifiers such as light exposure, oxidative stress, inflammation, and aging, which can remodel chromatin and increase transcriptional noise over time. Clinically, these mechanisms provide a plausible substrate for variability in onset and progression within families and across genetically homogeneous cohorts.

From a translational standpoint, epigenetic heterogeneity may help explain variable responses to gene augmentation, RNA therapies, and genome editing, because restored genes still require a permissive chromatin environment to be expressed and integrated into existing regulatory networks.

For clarity, the principal epigenetic mechanisms implicated in IRDs, their associated regulators, evidence base, and translational considerations are summarized in Table 1.

**TABLE 1:** Summary of epigenetic mechanisms implicated in inherited retinal disorders (IRDs), associated regulators, level of evidence, and translational considerations.

Mechanism	Key regulators	Retinal process	Example IRDs/syndromic disorders	Evidence level	Translational note
DNA methylation	DNMT1, DNMT3A/B, TET enzymes	Photoreceptor lineage specification; maintenance of neuronal identity; stress-response regulation	Retinitis pigmentosa (RHO, RPGR); leber congenital amaurosis (CRX-associated forms)	Primarily animal models; limited human tissue data; organoids	DNMT/TET modulation potentially druggable; risk of global epigenomic instability and off-target effects
Histone acetylation/deacetylation	HDAC1–3, class I/II HDACs; HATs (CREBBP, EP300)	Photoreceptor differentiation; chromatin accessibility during degeneration; metabolic gene regulation	Retinitis pigmentosa; rubinstein–Taybi syndrome	Extensive preclinical data; limited direct human retinal validation	HDAC inhibitors show neuroprotection in models; concerns: cell-type specificity, systemic toxicity, dose optimization
Histone methylation (e.g., H3K4, H3K27)	PRC2 complex; KMT2D; KDM6A	Retinal progenitor proliferation; enhancer activation; transcriptional stability	Kabuki syndrome; developmental retinal dystrophies	Genetic syndromic evidence in humans; mechanistic studies in models	Targeting methyltransferases/demethylases is complex; high risk of broad transcriptional disruption
ATP-dependent chromatin remodeling	SWI/SNF-related complexes; chromatin remodelers (e.g., ARID family)	Higher-order chromatin organization; stress-adaptive transcription	Syndromic retinal disorders; neurodevelopmental syndromes with retinal involvement	Mostly experimental models; rare human genotype–phenotype correlations	Limited pharmacologic specificity; systemic effects likely
Non-coding RNAs (miRNAs)	Dicer; miR-183/96/182 cluster; retina-enriched miRNAs	Photoreceptor differentiation; post-transcriptional buffering; oxidative stress modulation	ABCA4-associated cone–rod dystrophy; RP models	Animal models; retinal organoids; some human expression profiling	RNA-based modulation feasible; delivery and stability remain major challenges
Cell-type–specific epigenomic states	Enhancer landscapes; cell-specific chromatin accessibility patterns	Rod vs. cone vulnerability; RPE metabolic regulation; stress responsiveness	Variable expressivity across IRDs	Single-cell epigenomics (human + organoids); largely descriptive	Potential for stratified therapy; requires precise targeting to avoid non-target effects

Epigenetic interventions are attractive because they can target convergent degeneration pathways and may be applicable across multiple genotypes (Popova et al., 2025). Nevertheless, translating epigenetic modulation to IRDs faces distinct challenges.

Target specificity and safety are central limitations. Most chromatin regulators control broad gene programs; systemic exposure or non-selective retinal targeting risks unwanted transcriptional reprogramming, with potential consequences for synaptic signaling, immune responses, and tumor-suppressor pathways. Strategies to improve specificity include localized ocular delivery, use of more selective enzyme inhibitors, and cell-type–restricted approaches (e.g., vectorized epigenome editors), but these remain largely preclinical.

Delivery and pharmacokinetics remain unresolved. Achieving effective concentrations in outer-retinal photoreceptors or RPE is challenging; intravitreal dosing favors inner retina, whereas subretinal delivery offers better outer-retinal access but is more invasive. For chronic diseases, durability is also a concern: transient modulation may require repeated dosing, while prolonged modulation increases the risk of ‘overshoot’ and persistent off-target effects.

Timing and disease stage likely determine benefit. Early intervention may exploit greater epigenetic plasticity, while late-stage disease may feature fixed chromatin states and extensive cell loss, limiting the ceiling of functional rescue even if stress pathways are normalized. This has practical implications for trial design and endpoint selection in IRDs.

Combination strategies are promising but require caution. In principle, epigenetic modulation could enhance transcriptional competence and improve expression of delivered transgenes, or stabilize stressed cells to extend the therapeutic window for gene replacement/editing. Conversely, global chromatin modulation might unpredictably alter promoter activity, immune signaling, or transgene variability across cell populations.

Finally, biomarkers and patient stratification will be important for clinical translation. Single-cell and multi-omic atlases increasingly enable identification of cell-type–specific chromatin states and stress signatures, which could help select patients with preserved transcriptional plasticity and define pharmacodynamic readouts for epigenetic drugs (Cowan et al., 2020; Wang et al., 2022; Liang et al., 2023; Finkb et al., 2022).

## 5 Conclusion

Epigenetic regulation is integral to retinal development, long-term neuronal identity, and stress adaptation. Evidence from IRDs and syndromic disorders supports a model in which chromatin state can modify disease severity and therapeutic responsiveness. While epigenetic therapies may offer mutation-independent neuroprotection, their clinical translation will require improved targeting, rigorous safety assessment, and integration with gene- and cell-based interventions.
